# Is vascular insulin resistance an early step in diet-induced whole-body insulin resistance?

**DOI:** 10.1038/s41387-022-00209-z

**Published:** 2022-06-08

**Authors:** Lauren Carmichael, Michelle A. Keske, Andrew C. Betik, Lewan Parker, Barbara Brayner, Katherine M. Roberts-Thomson, Glenn D. Wadley, D. Lee Hamilton, Gunveen Kaur

**Affiliations:** 1 Institute for Physical Activity and Nutrition (IPAN), School of Exercise and Nutrition Sciences, Deakin University, Geelong, Australia; 2 Menzies Institute for Medical Research, University of Tasmania, Hobart, Tasmania Australia

**Keywords:** Pre-diabetes, Pre-diabetes

## Abstract

There is increasing evidence that skeletal muscle microvascular (capillary) blood flow plays an important role in glucose metabolism by increasing the delivery of glucose and insulin to the myocytes. This process is impaired in insulin-resistant individuals. Studies suggest that in diet-induced insulin-resistant rodents, insulin-mediated skeletal muscle microvascular blood flow is impaired post-short-term high fat feeding, and this occurs before the development of myocyte or whole-body insulin resistance. These data suggest that impaired skeletal muscle microvascular blood flow is an early vascular step before the onset of insulin resistance. However, evidence of this is still lacking in humans. In this review, we summarise what is known about short-term high-calorie and/or high-fat feeding in humans. We also explore selected animal studies to identify potential mechanisms. We discuss future directions aimed at better understanding the ‘early’ vascular mechanisms that lead to insulin resistance as this will provide the opportunity for much earlier screening and timing of intervention to assist in preventing type 2 diabetes.

## Introduction

Glucose homoeostasis is maintained by the actions of various tissues such as the brain, skeletal muscle, kidneys, blood cells, splanchnic organs and adipose tissue [1, 2]. However, the vasculature is another tissue required for optimal glucose and hormone delivery to target tissues [3]. Blood flow through the capillaries in skeletal muscle, also known as microvascular blood flow (MBF), is particularly important because it is responsible for the exchange of glucose, insulin and other nutrients from the blood and/or plasma to the myocyte [4]. In healthy individuals, skeletal muscle MBF increases in response to insulin or following a meal which facilitates glucose disposal [5]. In contrast, this vascular process is impaired during states of insulin resistance which is evident in obesity and type 2 diabetes (T2D) and leads to reduced muscle glucose uptake [6–8]. Therefore, impaired muscle MBF is an important contributor to skeletal muscle insulin resistance and increases the risk of pre-diabetes and T2D.

In pathological conditions such as overweight, obesity and T2D, individuals have impaired insulin and post-meal MBF responsiveness [9–11]. These groups often have elevated circulating free fatty acids (FFAs) [7] and ectopic fat deposition in various tissues [12, 13] and throughout the vasculature [11] which may contribute to impaired MBF and insulin resistance [14]. Healthy animals fed a high-fat diet (HFD), even with moderately raised fat content (1.8-fold) or for a short duration (3 days), have reduced MBF responses to the insulin that occurs before the development of obesity or myocyte/whole-body insulin resistance [7, 15]. However, what is not clear is if diet-induced insulin resistance impairs skeletal muscle MBF in humans and if this occurs before the signs of increased adiposity or insulin resistance. A better understanding of the early vascular defects that lead to insulin resistance and glucose intolerance will provide the opportunity for much earlier screening and interventions to prevent T2D.

Diets such as high calorie (HC) and/or HFD present a valid model to study diet-induced insulin resistance [16–19]. In this review, we summarise what is known about the effects of short-term HC and/or HFD on insulin action, glucose metabolism and on MBF in humans. We also explore selected animal studies to highlight possible mechanisms. This review aims to identify important gaps in the current literature and provide direction for future research in order to develop successful dietary strategies to prevent insulin resistance and pre-diabetes resulting from HC and/or high-fat feeding.

## Role of skeletal muscle blood flow in glucose metabolism

The vascular system can be categorised into two subgroups, the macrovasculature and microvasculature. The macrovasculature is inclusive of larger branches of vessels consisting of the arteries and veins, whereas the microvasculature includes the smallest branches of vessels that form the capillary networks that are embedded within a tissue [4]. The capillaries are thin-walled (often single cell) which allows for the exchange of nutrients, hormones and gases. Insulin is a key player in skeletal muscle macrovascular blood flow, MBF and myocyte glucose uptake [20, 21]. However, changes in MBF are not always mirrored by comparable changes in macrovascular flow as some studies have reported increases in total blood flow without any changes in MBF and vice versa [21, 22]. These findings have been made possible by the development of a number of techniques to measure macro- and micro-vascular blood flow (detailed in Table 1 and extensively reviewed elsewhere [23]. Changes in macrovascular blood flow occur after insulin’s action on increasing glucose disposal in skeletal muscle, suggesting a temporal dissociation between total limb blood flow and muscle glucose metabolism [24]. Insulin-dependent increases in MBF precede that of total limb blood flow and can occur at lower insulin concentrations [25, 26]. Our research team have also shown that insulin-stimulated increases in MBF can occur independent of changes in macrovascular blood flow but changes in MBF are rapid, and therefore MBF may facilitate the early increase in glucose uptake [4, 26].

**Table 1:** Methods for measurements of blood flow.

Method	Summary	Advantages	Disadvantages
*Total blood flow*			
Venous occlusion plethysmography	Venous drainage from a limb, usually forearm is occluded using an inflated cuff and changes in volume are measured by a plethysmograph. The linear increase in the limb volume is directly proportional to total arterial blood flow.	-Simple and minimally invasive -Allows the study of local vascular physiology in the forearm without affecting systemic circulation	-Indirect measure of blood flow -Measurements need to be done in a short time period to avoid ischaemia due to occlusion -Measurements need to be done at rest -Does not allow for the collection of venous blood samples from the same limb
Thermodilution	Ice-cold saline is infused into the vein of a limb and blood temperature is dropped and regularly monitored at intervals using a resistance thermometer. Total blood flow is calculated using a heat balance equation.	-Can be used during exercise	-Mildly invasive technique -Does not allow for continuous measurement of blood flow
Doppler ultrasound	An ultrasound probe is placed on the artery of interest it transmits sound waves that are reflected by the moving erythrocytes in the blood which shifts the Doppler frequency. The frequency at which the sound wave is transmitted and received, the insonation angle, the speed of moving erythrocytes and the vessel cross-sectional area are all used in an equation to calculate the total blood flow.	-Non-invasive -Allows for continuous measurement of blood flow -Can be used during exercise such as one leg knee extension	-Requires expensive equipment and software -Requires good technical expertise and standardisation of probe angle -Movement can cause noise to the ultrasound signal
*Microvascular blood flow*			
Contrast-enhanced ultrasound (CEU)	CEU utilises the infusion of gas-filled phospholipid or albumin microbubbles. Due to the nature of the size of the microbubbles, they are able to enter and stay within the entire vascular network. The microbubbles oscillate and enhance the ultrasound signal and can be destructed using a high-energy pulse. With a constant infusion, they reappear in vessels within the imaging beam. After background subtracting microbubble signal from arteries/veins and tissue per se, the rate of reappearance of the microbubbles within a region of interest provides a measurement of the microvascular re-filling rate (i.e. velocity), whereas the overall acoustic intensity is a measure of microvascular blood volume. The net microvascular blood flow is calculated as the product of microvascular blood volume and microvascular flow velocity.	-Allows for assessment of microvascular blood flow in tissues such as skeletal muscle, adipose, heart, kidney and liver	-Requires expensive equipment and software -Is limited by the number of microbubbles that can be infused in a participant -Requires good technical expertise and standardisation of ultrasound settings and probe position
1-methylxanthine (1-MX) infusion	This technique involves infusion of 1-methylxanthine (1-MX) which is metabolised to 1-methylurate (1- MU) by microvascular xanthine oxidase. Xanthine oxidase is located primarily on capillary endothelial cells, and not on large arteries and veins, or in myocytes. 1-MX and 1-MU can be quantified in plasma using high-performance liquid chromatography. The disappearance of 1- MX across the limb (A- V difference x arterial blood flow) is used as a biochemical marker for the extent of muscle microvascular surface area.	-Allows for assessment of microvascular blood flow in skeletal muscle	-Xanthine oxidase activity is not as high in humans as in rodents. This method is limited to rodent studies and only single time point.
Near-infra-red spectroscopy (NIRS)	NIRS method uses a light source emitting two or more wavelengths of light in the near-infra-red range into the tissue of interest and a detector placed at a known distance from the source(s). The oxygenated and deoxygenated haemoglobin absorbs infra-red light differently and their contribution to NIIRS infra-red signal allows for the assessment of skeletal muscle hemodynamics.	-allows assessment of microvascular blood flow -is non-invasive -Can be used to study oxygen consumption by the muscle	-Signal can be affected by the thickness of skin and adipose tissue. -Velocity cannot be separated from volume; thus no information on capillary perfusion is available
Laser doppler flowmetry (LDF)	LDF technique uses the assessment of the Doppler shift of low-power laser light, which is scattered by moving red blood cells to estimate blood flow.	-non-invasive -allows assessment of microvascular blood flow	-No absolute values -No depth information
Positron emission tomography (PET)	PET method involves intravenous injection of a radiolabelled tracer and the radioactivity emitted by the tracer is followed over time by a PET scanner within the region of interest. The kinetics of the tracer is then used to calculate the magnitude of blood flow.	-allows assessment of microvascular blood flow -Can use various tracers to study metabolisms such as labelled water or glucose and also study oxygen consumption by the muscle	-Requires expensive scanner

Insulin-dependent increases in skeletal muscle MBF are regulated by the balance of nitric oxide (a potent vasodilator) and endothelin-1 (a potent vasoconstrictor) which are produced via complex biochemical pathways [4]. Nitric oxide is produced by the endothelial cells in response to insulin, and diffuses into the adjacent smooth muscle cells, causing relaxation and resulting in vessel dilation and increased capillary surface area of downstream capillaries [4]. As a result, glucose disposal to the tissues is enhanced due to increased delivery of glucose and insulin to the myocyte. Insulin-stimulated microvascular perfusion is seen whether insulin is secreted from the pancreas (e.g. during a mixed meal challenge) or via exogenous insulin infusion (e.g. during hyperinsulinemic–euglycemic clamp) [4]. Blocking insulin-mediated increases in muscle MBF leads to a reduction of muscle-specific glucose disposal by ~40% when assessed using the hyperinsulinemic–euglycemic clamp technique [27], which provides evidence of the role of MBF in glucose disposal. Insulin’s microvascular actions are acutely blunted with vasoconstrictive agents (e.g. α-methylserotonin [28] and endothelin-1 [29]) and inflammatory cytokines (e.g. tumour necrosis alpha [30]) which consequently causes insulin resistance. Importantly, the infusion of vasodilators during hyperinsulinemic conditions does not always overcome microvascular insulin resistance or improve insulin sensitivity and glucose disposal in muscle despite increased macrovascular blood flow [31]. This indicates that macrovascular blood flow is sometimes permissive rather than stimulatory for glucose disposal. For example, vasodilation with methacholine in the presence of insulin in rats stimulates MBF and skeletal muscle glucose uptake, whereas, a similar degree of vasodilation with bradykinin does not [32]. Treatments that augment insulin-stimulated MBF also improve insulin sensitivity and these include: metformin [33], 5-aminoimidazole-4-carboxamide-1-β-D-ribofuranoside (activator of AMP-activated protein kinase) [34], glucagon-like peptide-1 [35], sodium salicylate [36] and exercise training [37, 38]. Therefore, it is well established that impaired skeletal muscle MBF is a contributor to insulin resistance, and improvements in MBF can augment insulin sensitivity. Understanding what factors (including diet) contribute to microvascular insulin resistance is important for preventing the development of pre-diabetes and T2D.

### Effect of short-term high calorie and/or high-fat diet in inducing insulin resistance

The literature consistently reports that short-term HC, HC with high-fat (HCHF) or HFD impair insulin action as measured by glucose tolerance tests, mixed meal challenges or hyperinsulinemic–euglycemic clamp (Table 2), confirming that high-fat and/or HC diets are a suitable model to study diet-induced insulin resistance. For example, Durrer et al. [39] showed a 17% increase in glucose area under the curve during an oral glucose tolerance test (OGTT) after 7 days on an HFD. Morrison et al. [40] reported a 14% increase in post-prandial glucose area under the curve and a 31% increase in post-prandial insulin area under the curve following a mixed meal challenge after 28 days on an HC diet. Similarly, a 7-day HCHF feeding in both men and women also increases the post-prandial glucose area under the curve and insulin area under the curve in response to a mixed meal challenge [19, 41]. The increased area under the curve for glucose and insulin suggests glucose intolerance alongside insulin resistance as a result of overfeeding. Insulin resistance has also been demonstrated by an increased homoeostatic assessment model of insulin resistance (HOMA-IR) in as early as 5 days [42] and 7 days [41, 43–45]. Reduced insulin sensitivity as a result of HC and HCHF feeding has also been identified using the hyperinsulinemic–euglycemic clamp technique [17, 45–47].

**Table 2:** Summary of human studies investigating effects of short-term high-calorie and/or high-fat diet on metabolic outcomes.

Study		Study details			Outcomes of interest			
	Duration	Participants	Type of diet	Intervention details	Body weight	Fasting glucose and fasting insulin	Glucose tolerance or insulin sensitivity	Blood flow
Cornier et al. [16]	3 days	13 (6 M/7 F) 9 reduced-obese (4 M/5 F)	HC	**50% increase in TE** Intervention: hyperenergetic 50% from CHO, 30% from FAT	No change	Not reported	No change in insulin sensitivity was assessed via HEC. The glucose reappearance rate was reduced by ~20% in lean women.	Not assessed
Cahill et al. [43]	7 days	*N* = 25 M normal weight (NW) *N* = 14 M overweight (OW) *N* = 25 M obese	HC	**70% increase in TE** Intervention: hyperenergetic diet (22.9 MJ, 50% CHO, 35% FAT)	Body weight increased by 2.1 kg in NW, 1.6 kg in OW 2.5 kg in obese Body fat increased by 0.14-0.8 kg	No change in fasting Glu, Fasting Ins increased by 45% in NW, 33% in OW and by 35% in obese	Increase insulin resistance by 46% in NW as assessed by HOMA-IR, 25% in OW and 31% in obese.	Not assessed
Schmidt et al. [73]	3 days	*N* = 22 (8 M/14 F) Obese prone *N* = 30 (16 M/14 F) Obese resistant	HC	**40% increase in TE** Intervention: hyperenergetic diet (46% from CHO, 35% from FAT)	No change	Not assessed	Not assessed	Not assessed
Wadden et al. [44]	7 days	*N* = 72 M	HC	**70% increase in TE** Intervention: hyperenergetic diet (22.8 MJ, 50% from CHO, 35% from FAT)	Body weight increased by 2.21 kg	No change in fasting Glu, Fasting Ins increased by 55%	Increase insulin resistance by 22.6% as assessed by HOMA-IR.	Not assessed
Boden et al. [45]	7 days	*N* = 6 M	HC	**150% increase in TE** Intervention: Hyperenergetic diet (25 MJ, 50% from CHO, 35% from FAT)	Body weight and body fat increased by 3.5 kg post 7 days	No change in fasting Glu, Fasting Ins by 150%	HOMA-IR increased by 166%. Reduced insulin sensitivity as assessed via 50% reduction in GIR during HEC.	Not assessed
Morrison et al. [40]	5 days and 28 days	*N* = 8 M	HC	**45% increase in TE** Intervention: hyperenergetic diet (+5 MJ, 55% from CHO, 30% from FAT)	5d: No change 28d: Body weight increased by 1.6 kg	5d: No change in fasting Glu, Increased fasting Ins by 15.9% 28d: No change in fasting Glu or Ins	5d: No change in 0-120 min AUC for Glu or Ins during an MMC 28d: Increase in AUC for Glu by 13.8% and Ins by 30.9% during an MMC	Not assessed
Emanuel et al. [51]	Average 29 days	*N* = 15 M Intervention	HC	**60% increase in TE** Intervention: hyperenergetic diet (60% from CHO and 25% from FAT)	Body weight and body fat increased by 3.5 kg	No change in fasting Glu or Ins	No change in insulin sensitivity was assessed via HOMA-IR and via HEC.	Significant increase in insulin-induced adipose tissue microvascular perfusion However, insulin-induced microvascular perfusion in muscle was impaired.
Dirlewagner et al. [50]	3 days	*N* = 10 F	HCHF	**40% increase in TE** Intervention: isoenergetic diet (7.5 MJ, 50% from CHO, 35% from FAT) Intervention: ↑CHO hyperenergetic diet (10.3 MJ, 64% from CHO, 25% from FAT) Intervention: ↑FAT hyperenergetic diet (10.5 MJ, 35% from CHO, **55% from FAT**)	Not reported	No change	No change	Not assessed
Keogh et al. [62]	21 days	*N* = 40 (19 M/21 F)	HCHF	**19% increase in TE** Intervention ↑PUFA: hyperenergetic diet (8.4 MJ, 45% from CHO, 36% from FAT [15% PUFA]) Intervention ↑MUFA: hyperenergetic diet (8.3 MJ, 44% from CHO, 37% from FAT [19% MUFA]) Intervention ↑CHO: hyperenergetic diet (8.0 MJ, 65% from CHO, 18% from FAT) Intervention ↑SFA:hyperenergetic diet (8.4 MJ, 45% from CHO, **37% from FAT** [19% SFA])	No change	Fasting Glu not reported No change in fasting Ins	Not assessed	Brachial artery FMD was reduced by 50% in the SFA group compared with ↑PUFA, MUFA and CHO groups.
Adochio et al. [48]	5 days	*N* = 21 (11 M/10 F)	HCHF	**40% increase in TE** Control: isoenergetic diet (50% from CHO, 30% from FAT) Intervention: hyperenergetic ↑CHO diet (60% from CHO, 20% from FAT) Intervention: hyperenergetic ↑FAT diet (30% from CHO, **50% from FAT**)	No change	No change in fasting Glu Fasting Ins increased in ↑CHO but not in ↑FAT group	No change in insulin sensitivity as assessed via HEC clamp	Not assessed
Brons et al. [46]	5 days	*N* = 26 M	HCHF	**50% increase in TE** Baseline: 11.8 MJ, 50% from CHO, 35% from FAT) Intervention: hyperenergetic ↑FAT diet (17.7 MJ, 32.5% from CHO, **60% from FAT**)	No change	Increased fasting Glu by 10% No change in fasting ins	Increased AUC from 0-30 mins for insulin (but not glucose) during an IVGTT. Two-fold increase in hepatic insulin resistance but no effect on glucose uptake as assessed via HEC.	Not assessed
Tam et al. [17]	28 days	*N* = 36 (17 M/19 F)	HCHF	**44% increase in TE** Baseline: 9.4 MJ, 55% from CHO, 30% from FAT Intervention: hyperenergetic ↑FAT diet (13.5 MJ, 40% CHO, **45% FAT**)	Body weight increased by 2.7 kg and body fat by 1.1 kg	Fasting glucose increased by 2.2% and fasting insulin by 15% post ↑FAT.	11% reduction in insulin sensitivity as assessed by HEC.	Not assessed
Bakker et al. [47]	5 days	12 M South Asian 12 M Caucasian	HCHF	**55% increase in TE** Intervention: hyperenergetic ↑FAT diet (+5.3 MJ, 32% from CHO, **54% from FAT**)	No change	Increased fasting Glu (by 20%) and Ins (by 49%) in South Asians No change in Caucasians	20% reduction in insulin sensitivity reduced in South Asians only as assessed via HEC clamp.	Not assessed
Boon et al. [42]	5 days	12 M South Asian 12 M Caucasian	HCHF	**55% increase in TE** Intervention: hyperenergetic ↑FAT diet (+5.3 MJ, 32% from CHO, **54% from FAT**)	Body weight increased by 0.5 kg	Increase in fasting Glu by 4% and increase in fasting Ins by 55% after 5 days	47% increase insulin resistance as assessed by HOMA-IR.	Not assessed
Parry et al. [19]	7 days	*N* = 9 (5 M/5 F)	HCHF	**50% increase in TE** Intervention: hyperenergetic ↑FAT diet (22% from CHO, **64% from FAT**)	Body weight increased by 0.79 kg	Increased fasting Glu (by 5%) No change in fasting insulin	Increase AUC for Glu by 11.6% and Ins by 25.9% during MMC.	Not assessed
Parry et al. [41]	7 days	*N* = 15 (13 M/2 F)	HCHF	**47% increase in TE** Intervention: hyperenergetic ↑FAT diet (19.8MJ, 20% from CHO, **64% from FAT**)	Body weight increased by 1.32 kg	Fasting Glu increased by 3.9% and fasting Ins by 19.4%	Increased AUC for Glu by 11% and Ins by 19% during an MMC. 24% reduction in insulin sensitivity as assessed by HOMA-IR.	Not assessed eNOS content within terminal arterioles reduced by 6%, no change in eNOS within capillaries.
Lundsgaard et al. [74]	3 days	*N* = 8 Medium-chain SFA N = 9 Long-chain SFA	HCHF	**75% increase in TE** Intervention 1: hyperenergetic ↑MCSFA FAT diet (+75% MJ, **82% from FAT** [5% TE medium chain FA]) Intervention 2: hyperenergetic ↑LCSFA FAT diet (+75% MJ, **82% from FAT** [5% TE long chain FA])	Not reported	Postabsorptive Glu increased by 9% and Ins by 77% with LCSFA. No change with MCSFA	21% reduction in insulin sensitivity and 17% in glucose disposal as assessed via HEC in the LCSFA group.	Not assessed
Wardle et al. [52]	6 days	*N* = 10 M HCHF-C *N* = 10 M HCHF-FO	HCHF	**150% increase in TE** Intervention HCHF-C: hyperenergetic ↑FAT diet (25% from CHO and 60% from FAT) Intervention HCHF-FO: hyperenergetic ↑FAT diet (25% from CHO and **60% from FAT** [6% TE from fish oil])	Body weight increased by 0.5 kg in HCHF-C and 1 kg in HCHF-FO	Not reported	No difference in HOMA-IR. No change in Glu or Ins during OGTT.	Not assessed
Whytock et al. [75]	7 days	*N* = 11 M, 11 F	HCHF	**50% increase in TE** Intervention: hyperenergetic ↑FAT diet (**65% from FAT**)	No change	No change in fasting Glu or Ins	No change in AUC for Glu or Ins during an OGTT	Not assessed No change in arterial stiffness
Anderson et al. [49]	5 days	*N* = 6 M	HFD	Baseline: isoenergetic diet (55% from CHO, 30% from FAT) Intervention: isoenergetic ↑FAT diet (30% from CHO, **55% from FAT**)	No change	No change	No change in insulin resistance assessed via HOMA-IR.	Not assessed
Durrer et al. [39]	7 days	*N* = 9 M	HFD	Baseline: isoenergetic diet (46% from CHO, 37% from FAT) Intervention: isoenergetic ↑FAT diet (11% CHO, **71% from FAT**)	Not reported	Not reported	Increase in AUC for Glu ~17% during an OGTT No change in Ins during OGTT.	↑FAT diet reduced the fasting FMD (−0.71%) but no effect on cerebral blood flow

*M* male, *F* female, *TE* total energy, *HFD* high fat (>35% of energy from fat), *HCHF* high calorie high fat, *HC* high calorie (with ≦35% energy from fat), *CHO* carbohydrate, *PUFA* polyunsaturated fat, *SFA* saturated fat, *Glu* glucose, *Ins* insulin, *OGTT* oral glucose tolerance test, *IPGTT* intraperitoneal glucose tolerance test, *ITT* insulin tolerance test, *MMC* mixed meal challenge, *AUC* area under the curve, *HEC* hyperinsulinemic– euglycemic clamp, *HOMA-IR* Homoeostatic model assessment of insulin resistance, *eNOS* endothelial nitric oxide synthase, *FMD* flow-mediated dilation.

High-calorie and/or high-fat intervention highlighted in bold.

Some studies report no changes in insulin action with overfeeding [48–52]. These inconsistent findings could be due to a large variation in the overfeeding protocols used in different studies. For example, studies with HC interventions have increased energy intakes ranging from +40% to +150%, and studies with HF interventions have total fat intakes ranging from 37% and 82% of total energy (Table 2). This wide range of overfeeding protocols may lead to differing metabolic loads and contribute to inconsistent glucose and insulin outcomes. There are also other limitations in regard to study design including small sample sizes in some studies (for example some studies only investigated 6 participants) [45, 49], large variations in the age range of participants (Bakker et al. [47] investigated 19 to 26-year-old participants, whereas Boden et al. [45] investigated 46 to 55-year-old participants), uneven distribution of males to females (with many studies predominantly recruiting males [40, 42–47, 51]), all of which are factors that may have contributed to variations in study outcomes. Nonetheless, most studies do suggest that short-term HC/HCHF/HF diets induce glucose intolerance and insulin resistance in humans.

#### Mechanisms for impaired glucose metabolism and insulin action

Both human and animal studies suggest various mechanisms by which short-term overfeeding can impair glucose tolerance and insulin sensitivity. One of these mechanisms involves increased circulating FFAs, greater adiposity and ectopic fat deposits in skeletal muscle and the vasculature per se. Boden et al. [45] reported an increase in both body weight and total body fat by 3.5 kg after 7 days of HC feeding (+150% calories with 35% of total energy from fat). Increases in calories or fat consumption are linked to hyperplasia and hypertrophy of the adipocytes thereby causing increased fat mass and weight gain [53]. Hypertrophic adipocytes exhibit reduced blood flow leading to a greater hypoxic and inflammatory cellular environment [54]. The inflammation also results from increased macrophage infiltration of the adipose tissue and dysfunctional cytokine/adipokine production [55] which include leptin, tumour necrosis alpha, interleukin-6, interleukin-8, interleukin-1 and monocyte chemoattractant protein-1. The increased inflammatory environment may cause insulin resistance and reduced glucose uptake in insulin-sensitive tissues (muscle and liver), thereby causing glucose intolerance.

Lipid accumulation in the skeletal muscle as a result of overfeeding may be another mechanism for impaired glucose metabolism and insulin action. Andrich et al. [56] reported that after 14 days of HFD (61% of total energy) the percent area and the average size of intramyocellular lipid droplets were significantly increased in the soleus muscle of HFD-fed rats. In line with these results, Wardle et al. [52] reported that a HCHF diet (150% energy with 60% total energy from fat) for 6 days significantly increased the accumulation of ceramides by 1.4 fold in the skeletal muscle in humans [52]. Skeletal muscle ceramide content is closely linked with insulin resistance in skeletal muscle [57]. Boon et al. [42] reported that insulin resistance induced by 5 days of HCHF feeding (as assessed by HOMA-IR) also increased the expression of various macrophage markers (for example cluster of differentiation CD68, CD14 and CD11c) in skeletal muscle and reduced the markers of insulin signalling (solute carrier family transporter SLC2A and glycogen synthase-1). Therefore a disruption in the insulin signalling pathway could be another mechanism for diet-induced insulin resistance. A limitation of this study is the investigation of mRNA expression of insulin signalling genes which are not always reflective of protein function and enzyme activity.

Oxidative stress induced by overfeeding can also disrupt insulin signalling [45]. Oxidative stress is associated with several glucose transporter-4 posttranslational modifications in particularly carbonylation (alteration of protein function) which may lead to impaired insulin-stimulated glucose transport [45]. Boden et al. [45] showed that oxidative stress inhibits insulin signalling by inactivating insulin receptor substrate 1/2 and Adocchio et al. [48] identified increases in skeletal muscle serine phosphorylation of insulin receptor substrate-1 in healthy participants who were given an HCHF diet (40% increase in energy with 50% of total energy from fat) for 5 days. Degradation (by 35%) of the intracellular insulin receptor was demonstrated after 10 days of HFD (67% of total energy) in rodents alongside reduced muscle glucose uptake [58].

Overall, despite short-term overfeeding inducing minor weight gain, it does not consistently impair fasting glucose and/or fasting insulin levels. However, overfeeding does impair functional outcomes of glucose tolerance and insulin action in humans as measured postprandially or during hyperinsulinemic–euglycemic clamp. However, it is not known if diet-induced insulin resistance impairs skeletal muscle blood flow in humans.

### Effect of short-term high calorie and/or high-fat diet on skeletal muscle blood flow

#### Macrovascular blood flow

The literature investigating the effects of a short-term HC, HFD and/or HCHF feeding on the large artery or macrovascular blood flow in humans is sparse. Bui et al. [59] reported that the ingestion of a single high-fat meal (with 50 g of fat), compared to a low-fat meal (with 5.1 g of fat), significantly reduced total forearm blood flow (19.3%) as measured by venous occlusion plethysmography in healthy participants. Flow-mediated dilation (FMD) is an indicator of vascular endothelial health and has been investigated in acute high fat meal ingestion studies (single meal) [60, 61] and short-term HFD studies [39, 62]. Single high-fat meal studies report no change in FMD with the amount of fat ranging from 50–90g [60, 61]. Durrer et al. [39] showed reduced FMD and impaired glucose tolerance after 7 days of HFD (71% of total energy) in healthy participants. Keogh et al. [62] showed that HFD (with high saturated fat) reduces FMD by 50% within 3 weeks. Overall, despite limited research on the effect of short-term overfeeding on macrovascular blood flow in humans, studies looking at large artery dilation suggest that a short-term HFD may impair endothelial function. The reduced endothelial function has been linked to reduced insulin-mediated nitric oxide production and reduced muscle glucose uptake [3].

#### Microvascular blood flow (MBF)

It is known that individuals who have raised plasma FFA levels become insulin-resistant and have impaired skeletal muscle MBF responses to insulin [6, 63]. Several studies have investigated the effects of lipid infusion on MBF in humans and report impaired insulin-mediated MBF responses [63–66]. For example, one of the studies used lipid infusion (Intralipid plus heparin) to raise plasma FFAs, and investigated subsequent effects on insulin (*n* = 23) and meal (*n* = 10) related MBF and compared it to saline infusion as a control [6]. The authors showed that 3 h after saline infusion, both mixed meal challenge and insulin infusion (hyperinsulinemic–euglycemic clamp) increased insulin levels and stimulated MBF, as measured using the contrast-enhanced ultrasound (CEU) method (Table 1 for method details). However, 3 h after lipid infusion (which raised plasma FFA by 18-fold) MBF was blocked during both the mixed meal challenge and the insulin clamp despite an increase in insulin concentrations. The authors also observed decreased forearm insulin-stimulated glucose disposal (during the clamp) and elevated plasma glucose during the mixed meal challenge demonstrating insulin resistance [6]. This study provides evidence for a link between circulating lipids and impaired skeletal muscle microvascular function. However, lipid infusion is not a physiological model, and as highlighted in Table 2, there is a significant gap in the literature regarding the effects of diet-induced insulin resistance on skeletal muscle MBF in humans.

To our knowledge, there is only one human study that looked at the effect of HC feeding on skeletal muscle MBF [51]. This study fed healthy men a HC diet (60% increase in calories with 25% from fat) for an average of 29 days. The HC diet increased body weight by 3.5 kg but there was no change in fasting glucose, insulin or insulin sensitivity as assessed via hyperinsulinemic–euglycemic clamp [51]. However, the authors reported an impairment in the normal insulin-mediated increase in muscle MBF measured by CEU. Interestingly, the MBF in adipose tissue increased suggesting the body is directing the excess nutrients to adipose for storage and protecting muscle from insulin resistance [51]. The fact that the insulin sensitivity was not altered suggests that vascular insulin resistance may be an early event that happens before whole-body insulin resistance. A time-course investigation in this model would be beneficial to confirm the timing of vascular versus muscle and whole-body insulin resistance.

In rodents, Premilovac et al. [7] and St-Pierre et al. [8] found that 4 weeks of HFD led to impairments in skeletal muscle MBF (assessed using the 1-methylxanthine method) and insulin sensitivity measured via hyperinsulinemic–euglycemic clamp. Kubota et al. [15] demonstrated that HFD-fed mice have impaired microvascular perfusion (assessed using CEU) in response to insulin and this coincided with whole-body and muscle-specific insulin resistance. The temporal association between MBF and glucose metabolism in rodents was demonstrated by Zhao et al. [67]. They found that an HFD (60% total energy from fat) in rodents reduced insulin-stimulated microvascular perfusion (assessed using the CEU method) in as early as 3 days which became progressively worse after 1, 2 and 4 weeks [67]. The corresponding impairments in whole-body glucose disposal were observed only after 1 week (and not 3 days) which suggests impairments in MBF occur before impairments in glucose disposal. As such, short-term high-fat feeding in rodents leads to impairment in MBF responses to insulin, and microvascular insulin resistance occurs before metabolic insulin resistance.

#### Mechanisms for impaired blood flow

It is suggested that elevated FFAs and the accumulation of lipid in tissues like muscle and liver leads to disruption in insulin signalling, causing insulin resistance and impaired endothelial function [68]. Protein kinase B (Akt) signalling in endothelial cells plays a crucial role in the regulation of vascular homoeostasis. It also stimulates the expression and activity of endothelial nitric oxide synthase (eNOS) and improves endothelial function [69]. Parry et al. [41] reported that 7 days of HC feeding (45% increase in total energy) reduced insulin-stimulated eNOS Ser-phosphorylation in terminal arterioles of skeletal muscle and reduced glucose clearance in healthy participants. Parry et al. [41] suggested that reduced eNOS may have reduced nitric oxide production thereby reducing skeletal muscle MBF causing impaired glucose metabolism, although they did not specifically measure blood flow. Zhao et al. using a rodent model showed that one week of HFD leads to insulin resistance during a hyperinsulinemic-euglycemic clamp, impaired muscle MBF (assessed via CEU), abolished insulin-stimulated Akt and eNOS phosphorylation and increased inflammation in the aorta but not in muscle [67]. When the authors pharmacologically reduced inflammation, the microvascular function was restored, suggesting that inflammation plays a role in the development of microvascular dysfunction in HFD-fed animals. Chai et al. [70] showed that 4 weeks of HFD (60% of total energy from fat) significantly blunted the insulin-mediated increase in plasma nitric oxide and increased the levels of plasma endothelin-1 which is a potent vasoconstrictor. Other animal studies show that hormones like glucagon-like peptide-1 (GLP-1) and the globular form of adiponectin can restore impaired muscle MBF impairments and improve glucose uptake via nitric oxide-dependent mechanisms [71, 72].

**Fig. 1:**
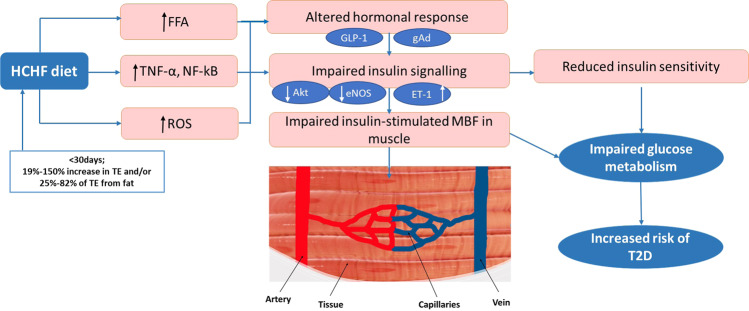
Mechanisms through which short-term high-calorie and/or high-fat feeding may impair muscle microvascular blood flow and insulin sensitivity in humans leading to impaired glucose metabolism. TE, total energy, HCHF, high-calorie high fat, FFA, free fatty acids, TNF-α, tumour necrosis alpha, NF-kB, nuclear factor kappa-light-chain-enhancer of activated B cells, GLP-1, glucagon-like peptide, gAd, globular adiponectin, Akt, protein kinase B, eNOS, endothelial nitric oxide synthase, ET-1, endothelin-1, MBF, microvascular blood flow, ROS, reactive oxygen species, T2D, type 2 diabetes.

Therefore, findings within animal models suggest that HFD leads to increases adipose tissue mass, and inflammation, reduces eNOS and nitric oxide production and increases endothelin-1 levels. All these factors impair insulin-mediated muscle MBF and thereby impair glucose homoeostasis (Fig. 1). Interestingly, the HFD-induced impairments in microvascular perfusion were of similar magnitude to the impairments in the endothelium specific insulin receptor substrate-2 knock-out model which also has impaired insulin signalling in the endothelium and reduced eNOS activation [15].

However, whether HC and/or HF feeding impairs insulin action in humans via impaired skeletal muscle MBF is still not clear. If these diets do impair MBF in healthy humans this would suggest there is a population group that is apparently “healthy” but at a high risk of developing insulin resistance and T2D. This group would be an ideal candidate for future dietary intervention and provide a driver for further investigation to prevent vascular insulin resistance caused by short-term HC and/or HF feeding.

## Conclusions and future directions

Current evidence supports a potential link between microvascular dysfunction and HC, HCHF or HFD-induced insulin resistance. However, the majority of evidence is derived from rodent research with very few studies conducted in humans. This is important to research further in humans, as vascular insulin resistance caused by overfeeding may occur before whole-body insulin resistance and the subsequent development of chronic diseases including T2D. A better understanding of the very early vascular defects that lead to insulin resistance and glucose intolerance in humans would provide the opportunity for earlier screening and appropriate interventions to prevent diet-induced T2D.

Current research on diet-induced insulin resistance in humans has some limitations such as the use of a wide range of dietary intervention protocols (% total energy and % of fat contribution to total energy), use of non-physiological methods of assessing glucose tolerance (eg. OGTT), use of methods that only measure large artery function and not muscle-specific MBF, and studies with an uneven male to female participant ratio. Nonetheless, one study in humans showed HC diet for 29 days impaired the normal insulin-mediated increase in muscle MBF but not insulin sensitivity. Future studies should use modern techniques in vascular imaging (e.g., CEU) with robust and physiologically relevant study design (e.g., mixed meal challenge rather than OGTT) with both male and female participants to advance this field of research. Analysis of blood/plasma and tissue biopsies from human participants with diet-induced insulin resistance is required to understand whether inflammation, oxidative stress and reduced nitric oxide synthesis are contributory mechanisms in humans. Confirming if vascular insulin resistance is an early step in whole-body insulin resistance in humans will be a major step forward in the field of targeting and designing strategies to prevent insulin resistance and T2D.
